# Maternal Serum SCUBE-1: A Novel Ischemic Marker in Preeclampsia

**DOI:** 10.3390/jpm14111102

**Published:** 2024-11-12

**Authors:** Gulseren Dinc, Suleyman Caner Karahan, Suleyman Guven

**Affiliations:** 1 Department of Obstetrics and Gynecology, Faculty of Medicine, Karadeniz Technical University, 61080 Trabzon, Turkey; sguven@ktu.edu.tr; 2 Medical Biochemistry, Faculty of Medicine, Karadeniz Technical University, 61080 Trabzon, Turkey; suleymancaner.karahan@ktu.edu.tr

**Keywords:** Doppler ultrasound, ischemia modified albumin, preeclampsia, SCUBE1

## Abstract

Background: SCUBE-1 (Signal peptide-CUB (complement C1r/C1s, Uegf, and Bmp1)-EGF (epidermal growth factor)-domain-containing protein 1) is a novel marker of ischemia, which is a cell surface-secreted protein in the platelets and endothelial cells. The aim of the study is to measure serum SCUBE-1 levels and investigate their association with uteroplacental blood flow in patients with preeclampsia. Methods: The study was conducted on patients with preeclampsia. Maternal serum SCUBE1 and IMA levels were the main outcomes. The control group consisted of gestational-age-matched pregnant women. Fetal umbilical artery (UA) pulsatility index (PI), middle cerebral artery PI, cerebroplacental ratio (CPR), and maternal uterine artery (UtA)-PI were also examined, and correlation analysis was performed to reveal the association between maternal serum SCUBE1 levels and Doppler findings. Results: The study group consisted of thirty-two preeclamptic patients, and the control group consisted of thirty-two uncomplicated singleton pregnancies. Maternal serum SCUBE1 and IMA levels were significantly higher in preeclamptic women compared to the control group (*p* < 0.000, *p* < 0.004, respectively). Mean UtA-PI values and fetal UA-PI values were significantly higher in preeclamptic pregnant women compared to the control group (*p* < 0.05, *p* < 0.05, respectively). However, the average CPR was significantly lower in pregnant women with preeclampsia (*p* < 0.05). While no significant correlation was found between maternal serum SCUBE1 levels and UA-PI and CPR (*p* > 0.05, *p* > 0.05, respectively), a significant correlation was found between right and left UtA-PI (*p* < 0.004, *p* < 0.006, respectively). Conclusions: The maternal serum SCUBE1 level is increased in patients with preeclampsia, and this increase is significantly correlated with the maternal uterine artery pulsatility index.

## 1. Introduction

Preeclampsia (PE), which affects 3–7% of all pregnant women, is one of the leading causes of maternal and fetal death, severe maternal and fetal morbidity, and maternal intensive care. Preeclampsia is a pregnancy complication that shows progressive multisystem involvement. It is basically a serious disease characterized by pregnancy over 20 weeks, hypertension with or without proteinuria, and/or some target organ dysfunction. The main pathophysiological mechanisms are abnormal placentation and maternal systemic vascular dysfunction. It is a disease that is likely to result in death if left untreated [1].

According to current international guidelines, the diagnostic criteria are the presence of a systolic blood pressure of 140 mmHg and above and/or a diastolic blood pressure of 90 mmHg and above when examined twice at least 4 h apart in cases of pregnancy over 20 weeks, plus one or more of the seven findings listed: (1) proteinuria ≥ 0.3 g in 24 h urine, or a ≥0.3 (30 mg/mmol) protein/creatinine ratio in random urine, or protein excretion ≥ 2+ in random urine (if quantitative measurement is not performed); (2) a platelet count < 100,000/µL; (3) a serum creatinine rate > 1.1 mg/dL, or in the absence of any kidney disease, a serum creatinine amount increasing twice compared to the pre-pregnancy level; (4) a liver transaminase enzyme level increasing at least twice the normal value limit; (5) the presence of pulmonary edema; (6) a new onset and persistent headache that does not respond to standard simple analgesics and cannot be explained by any other reason; and (7) vision problems (blurred vision, flashes of light, visual field defect) [2].

Common clinical risk factors are nulliparity, preeclampsia in a previous pregnancy, maternal obesity, maternal age >40 years or <18 years, family history of preeclampsia, chronic systemic disease (such as hypertension, kidney disease, autoimmune disease (e.g., antiphospholipid syndrome, systemic lupus erythematosus), vascular disease, diabetes mellitus (pregestational and gestational), hyperthyroidism, fetal factors (multifetal gestation, hydrops fetalis, fetal growth restriction, abruption, or fetal demise in a previous pregnancy), the patient themself was small for gestational age, prolonged interpregnancy interval (in cases where the previous pregnancy was normotensive), a short interpregnancy interval (in cases where the previous pregnancy included preeclampsia), male partner-related factors (such as new male partner, limited sperm exposure), assisted reproductive technology-associated pregnancies, sleep disordered breathing, elevated blood lead level, posttraumatic stress disorder, etc. In addition, there are publications showing that smoking and being of Asian or Hispanic origin reduces the risk of preeclampsia [2,3].

Preventive measures can be taken if risk factors are revealed at the first prenatal visit in a pregnant woman and if it is later determined that the patient has a high risk factor. In this context, it is partially possible to prevent preeclampsia if the patient started low-dose aspirin before the 16th week of pregnancy. According to the results of the current meta-analysis, it was reported that aspirin showed benefits depending on the week of initiation (initiating aspirin before rather than after 20 weeks of gestation (RR 0.85 at <16 weeks, RR 0.90 at 16 to 19 weeks, RR 0.99 at 20 to 23 weeks, RR 0.88 at 24 to 27 weeks, and RR 0.95 at ≥28 weeks) [4].

The definitive treatment for preeclampsia is delivery. After the placenta and fetus are delivered, the woman enters the recovery process. However, in cases of preeclampsia that develop in the early weeks of pregnancy, birth brings with it prematurity. This is an important issue for baby health. Therefore, if preeclampsia is predicted and appropriate precautions are taken, the longer the gestational age, the better the health of the baby and the mother will be [2]. Research has focused on the pathogenesis of preeclampsia, as the presence of effective markers that can predict preeclampsia will make an important contribution to the health of the mother and baby. In this context, research on the results of angiogenic/antiangiogenic factor balance in preeclampsia may be promising. This research on SCUBE-1 plans to fill this gap.

Many biochemical markers associated with hypoxia and endothelial dysfunction, including hypoxia-inducible factors (HIF)-2α, soluble fms-like tyrosine kinase 1 (sFLT1), transforming growth factor (TGF), vascular endothelin growth factor (VEGF), ischemia-modified albumin (IMA), etc., have been investigated in PE screening and etiopathogenesis [5]. IMA, an ischemia and oxidative stress marker, was first studied as a hypoxic/ischemic biomarker in cardiac patients; later, it has been shown that serum levels also increase in non-cardiac ischemic diseases [6,7,8,9]. It has also been shown that IMA increases in preeclamptic pregnant women [8,9].

SCUBE-1 (Signal peptide-CUB (complement C1r/C1s, Uegf, and Bmp1)-EGF (epidermal growth factor)-domain-containing protein 1) is a novel cell surface-secreted protein in the platelets and endothelial cells [10]. SCUBE-1 protein is mostly expressed in platelets and stored within the α-granules of inactive platelets and secreted under hypoxia and inflammatory conditions. In immunohistochemistry studies, diffuse SCUBE-1 deposition was detected in the subendothelial matrix of atherosclerotic plaques [11]. In the previous studies, it was shown that SCUBE-1 concentration was significantly elevated in acute coronary syndrome, myocardial hypoperfusion, acute ischemic stroke, and acute mesenteric ischemia [12]. Maternal serum SCUBE-1, which is a novel marker of ischemia, might have a potential role in diagnosis and screening of PE. To the best of our knowledge, when we searched the literature written in English, we did not find any studies investigating the relationship between serum SCUBE-1 levels and PE. The aim of this study is to investigate the relationship between PE and maternal serum SCUBE-1 levels and uteroplacental flow indices.

## 2. Materials and Methods

### 2.1. Study Design

The study was performed on pregnant women with PE who were followed up in our hospital within a 12-month period in 2020. Informed consent was obtained for all cases. The study methodology was approved by the hospital local ethics committee (Ethics Committee approval date 20 June 2020, number 2020-159). The principles of the Helsinki Declaration were complied with in the study. Pregnant women with chronic renal disease, heart disease, diabetes mellitus, chronic hypertension, thyroid dysfunction, hereditary thrombophilia, connective tissue disease, and multiple pregnancies and cases conceived by assisted reproductive methods were not included in the study. All pregnant women who were diagnosed with PE within 12 months and who volunteered to participate in the study constituted the study group, and uncomplicated singleton pregnant women with matched gestational weeks constituted the control group. The diagnosis of PE was made according to American College of Obstetricians and Gynecologists’ (ACOG’s) standard criteria published in 2013 [13]. Blood samples for the measurement of serum SCUBE1 and IMA levels was taken from pregnant women who were hospitalized and followed up with a diagnosis of PE.

### 2.2. Serum SCUBE1 and IMA Measurement

In order to measure serum SCUBE1 and IMA levels, 5 mL venous blood samples were taken from the forearms between 8.00 and 11.00 in the morning, in a serum separator gel containing vacutainer tubes, following an overnight fast. After the coagulated blood samples were centrifuged at 1200× *g* for 10 min, the obtained serum supernatants were stored at −80 °C for SCUBE-1 and IMA measurements. Serum SCUBE-1 concentrations were determined by using commercially available kits based on a sandwich-ELISA (Elabscience, Cat No: E-EL-H5405, Lot: Y5GV5ZEU8U, Hubei, Wuhan, China) method, and the results were expressed as ng/mL. The measurements of serum IMA levels were performed using the albumin-cobalt binding (ACB) colorimetric method based on Bar-Or et al. [14].

### 2.3. Doppler Examination

Ultrasonographic evaluation of all participants was performed via a Voluson E10 ultrasound system (General Electric’s Healthcare, Zipf, Austria) with a 4–8 MHz convex array transducer by a single specialist (M.O.) Fetal umbilical artery (UA), pulsatility index (PI). and mean cerebral artery (MCA)-PI, cerebroplacental ratio (CPR), and maternal uterine artery (UtA)-PI were studied on the day the blood sample was taken.

UA Doppler examinations were performed while the patient was placed in a semi-recumbent and left lateral tilt position. The UA Doppler waveforms were recorded from a free-floating segment or from several loops of umbilical cord segments and selected away from the cord insertion since the UA-PI is significantly higher at the fetal end of the cord than at the placental end. UA-PI was calculated from a minimum of three consecutive waveforms at an insonation angle <30° [15,16].

In the UtA Doppler examination, the probe was placed in the right lower quadrant of the abdomen and in color Doppler mode; the place where the uterine artery crosses the external iliac artery longitudinally was determined. Doppler flow forms of the UtA were recorded by placing a sample volume into a vessel from the segment before the uterine artery bifurcation and just 1 cm distal to the crossing point. A similar procedure was performed exactly for the left side [17].

The middle cerebral artery (MCA) was searched at the level of the sphenoid bone in axial sections where the thalamus and cavum septum pellucidum were seen at the level of the circle of Willis. The artery was colored in color Doppler mode when MCA pulses were seen. The sample volume was placed in the middle of the vessel, and the flow velocity waveforms were recorded by setting the insonation angle below 30°. Care was taken not to apply pressure to the fetal head during the examination, as pressure on the fetal head alters the flow velocity waveforms of the MCA. The CPR was calculated by dividing the MCA-PI by the UA-PI [15,16].

### 2.4. Statistical Analyses

SPSS 11.5 designed for Windows was used for the statistical analysis. All continuous variables were defined as mean and standard deviation, while categorical variables were defined as a percentage of the total group. All statistical tests were two-sided, and a *p*-value < 0.05 was determined as statistically significant. Student’s *t*-test and Fisher’s exact test were used to compare variables. The Spearman correlation test was used to determine the relationship between serum SCUBE1, serum IMA, and maternal and fetal Doppler findings.

## 3. Results

The study group consisted of 32 pregnant women with preeclampsia. Thirty-two uncomplicated singleton pregnancies constituted the control group. No statistical difference was found between the mean age, gravida, parity, body mass index (BMI), 5 min APGAR score, and gestational weeks. The mean newborn weight, and gestational age at delivery were significantly lower in the study group (Table 1).

The mean maternal serum SCUBE1 and serum IMA levels were significantly higher in preeclamptic women compared to the control group (*p* < 0.05). The mean maternal uterine artery PI and fetal umbilical artery PI were significantly higher in preeclamptic women compared to the control group (*p* < 0.001, *p* < 0.001, respectively). However, the average CPR values were significantly lower in preeclamptic pregnant women than the control group (*p* < 0.05), (Table 2).

The correlation between maternal serum IMA and SCUBE1 levels and maternal/fetal Doppler data is summarized in Table 3. There was no significant correlation between serum IMA levels and UA-PI, CPR, or UtA-PI values (*p* > 0.05, *p* > 0.05, *p* > 0.05, *p* > 0.05, respectively). While no significant correlation was found between maternal serum SCUBE1 levels and UA-PI and CPR (*p* > 0.05, *p* > 0.05, *p* > 0.05, respectively), a significant correlation was found between right and left UtA-PI (*p* < 0.004, *p* < 0.006, respectively).

## 4. Discussion

In the present clinical study, we showed that serum SCUBE1 and IMA levels increased in preeclamptic women. Additionally, we found that maternal serum SCUBE1 level is correlated with maternal uterine artery Doppler parameters, which is an indirect indicator of placental perfusion. This is the first clinical study revealing the association between the maternal serum SCUBE1 level and preeclampsia.

The human placenta forms extensive vascular networks during development. This is essential for the provision of nutrients necessary for the development of the baby. Many angiogenic factors are released by the placenta for proper placental angiogenesis. The most prominent proangiogenic factors are vascular endothelial growth factor (VEGF) and placental growth factor (PlGF). Apart from this, it has been reported that soluble fms-like tyrosine kinase-1 (sFlt-1) is released from the placenta as antiangiogenic factors. If the balance of proangiogenic factor and antiangiogenic factor is disrupted in favor of antiangiogenic factor, systemic endothelial dysfunction may occur. An increase in antiangiogenic factors such as sFlt-1 triggers placental-induced oxidative stress and hypoxia. This situation constitutes the basic physiopathology of preeclampsia [5].

sFlt-1 eliminates the angiogenic biological activities of circulating VEGF and PIF by binding them. According to human study results, it was reported that the sFlt-1 level was higher in preeclampsia cases, and VEGF and PIF levels were lower compared to normotensive cases [18]. Even in the early stages of pregnancy, the change pattern of these cytokines had been clearly demonstrated in cases that would develop preeclampsia [19]. Another current antiangiogenic marker that has a synergistic effect with sFlt-1 is soluble endoglin (sEng), which is a mediator released from the placenta that causes systemic endothelial dysfunction. According to the study results, serum levels increased two to three months before clinical preeclampsia findings occurred [20].

The investigation of maternal serum ischemia markers in the pathogenesis of PE is still up to date. In previous studies, it was shown that maternal serum IMA levels increased in preeclamptic women compared to healthy pregnant and non-pregnant women [6,7,8,21,22]. IMA increase in pregnant women with PE has been shown to be a simple and new marker for oxidative stress associated with PE [6,7,22]. The sensitivity of IMA in detecting PE was found to be between 0.75 and 0.88, and the specificity was between 0.44 and 0.91 [6]. The maternal serum IMA levels elevated in normal pregnancy were probably due to physiological oxidative stress in pregnancy [23]. In 2020, Karaşin et al. [7] studied maternal serum IMA levels in 30 preeclamptic pregnant women, and they found that serum IMA levels were higher in patients with PE compared with healthy pregnant women, but there was no statistically significant difference in terms of PE severity. They concluded that the lower concentration of maternal serum IMA in healthy pregnant women compared with preeclamptic patients suggests that inflammation and endothelial cell activity and oxidative stress in PE may increase the IMA levels. There are few studies comparing the severity of PE with serum IMA levels. Ustün et al. [24] studied serum IMA levels in 18 normotensive and 36 preeclamptic pregnant women. Patients were subdivided as having either mild or severe PE. They found that serum IMA levels were significantly higher in the mild and severe preeclamptic cases than in the control group [24]. D’Souza et al. [25] measured serum and saliva IMA and IMA–albumin ratio values in 50 preeclamptic (32 mild and 18 severe PE) and 50 normal pregnant women. They found that serum IMA levels significantly increased in PE, and the increase in serum IMA levels was in accordance with the severity of PE. As a result, IMA has been proposed as a predictive marker for PE. In our study, in accordance with the literature, serum IMA levels were found to be significantly higher in pregnant women with PE when compared with healthy pregnant women.

Similarly, many markers have been described in the literature showing the relationship between some new endothelial markers and uterine artery PI [26]. A recent study aimed to determine whether the combination of pregnancy-associated endothelial cell-specific molecule 1 (ESM-1), the placental growth factor (PLGF) in the first- and second-trimester maternal serum, and the uterine artery Doppler pulsatility index in the second trimester can predict preeclampsia. As a result of the study, the sensitivity and specificity of the combination of low ESM-1 levels in the first trimester, low PLGF levels in the second trimester, and high PI levels in the second trimester in terms of predicting preeclampsia were reported as 72.73% and 95.00% [27].

Hypoperfusion and ischemia are the main components in the pathogenesis of preeclampsia. Placental hypoperfusion causes secretion of a variety of factors into the maternal bloodstream that affect maternal endothelial function and lead to the characteristic systemic signs and symptoms of preeclampsia. SCUBE-1 is a novel cell surface protein predominantly stored within platelets and has been revealed by immunohistochemistry studies in the subendothelial matrix of advanced atherosclerotic lesions. It was demonstrated that SCUBE-1 is able to enhance platelet adhesion and agglutination; therefore, it may play pathological roles in cardiovascular pathology [12]. In this study, we found that maternal serum SCUBE-1 levels in preeclamptic women were significantly higher than normal pregnant women. Dai et al. [28] reported that plasma SCUBE-1 concentration is significantly elevated in acute ischemic stroke and acute coronary syndrome as early as 6 h after onset of ischemic symptoms and can be detected up to 84 h. Turkmen et al. [29] reported that SCUBE-1 levels were significantly higher in mesenteric ischemic patients within 6 h ischemia and have the potential to be used as markers in acute mesenteric ischemia. Uyanikoglu et al. [30] compared serum SCUBE-1 levels measured by ELISA in 15 patients with ovarian torsion and 20 healthy women of the same age. In their study, serum SCUBE1 levels were found to be significantly higher in patients with ovarian torsion compared to healthy women. Serum SCUBE-1 levels were found to be higher in patients with hypertension and myocardial infarction compared to normal healthy individuals [11,31].

High resistance/impedance indices including PI, RI, and S/D in both the UA and UtA have been shown to be associated with increased risk of PE [31,32]. Using the combination of uterine, umbilical, and MCA Doppler parameters (PI, RI, S/D, and CPR) is better at predicting PE than the components on their own. Abnormal MCA Doppler findings reflect fetal adaptation in hypoxia that develops in PE. On the other hand, the CPR was accepted as more accurate and more sensitive than PI and RI alone, and improved the detection of high risk adverse perinatal outcomes occurring in fetuses with normal umbilical artery Doppler [16,31,32]. Among the Doppler indices, PI was used in vascular impedance measurements in our study, since PI is generally accepted the best predictor for PE and adverse perinatal outcomes [31,32]. In the present study, UA-PI and UtA-PI were significantly different in PE compared with normal pregnancy. Additionally, although there was no difference between the MCI-PI values of both groups, CPR was found to be statistically significantly different between both groups. In the present study, while maternal serum SCUBE-1 values were found to be positively correlated only between uterine artery PI values, no significant correlation was found between UA-PI, CPR, and SCUBE-1. Additionally, no statistically significant correlation was found between serum IMA values and uterine, umbilical artery PI, and CPR. Therefore, maternal serum SCUBE-1 level in pregnant women with PE may be a useful marker that is more sensitive than IMA and can be used together with uteroplacental flow indices.

Screening for the development of preterm preeclampsia is important for mother and baby health. According to the fetal medicine foundation recommendation, attempts to predict preeclampsia are made by using a combination of parameters, such as maternal risk factors, the uterine artery Doppler pulsatility index, mean arterial pressure, and serum placental growth factor [11]. According to the results of another recent study, the week of pregnancy that resulted in a previous spontaneous birth was important in terms of the risk of early-week preeclampsia that may occur in the next pregnancy. The earlier in the previous pregnancy the birth occurs, the higher the risk of first trimester preeclampsia in the next pregnancy [33]. All these cause the ischemia-mediated preeclampsia hypothesis to come to the fore. Our study findings may suggest the serum marker associated with uteroplacental blood flow alterations in preeclamptic patients, providing a potential link between ischemic processes and Doppler findings. Therefore, new ischemia markers such as serum scube-1 are promising in predicting preeclampsia and protecting the health of the mother and baby. The results of this study may also open the door to new research on the pathogenesis of ischemia-mediated preeclampsia.

The most important limitation of the study is that it is a single center study, and the number of cases is low.

## 5. Conclusions

This is the first study showing the association between PE and maternal serum SCUBE-1 levels. In the present study, we have shown that SCUBE-1 is a novel ischemic marker, and its serum level increased in preeclamptic pregnant women. Additionally, a significant correlation was found between maternal serum SCUBE-1 levels and UtA-PI. Prospective studies are needed on the relationship between PE and uteroplacental flow indices and SCUBE-1, which appears to be a novel ischemic marker.

## Figures and Tables

**Table 1:** The maternal and neonatal characteristics of the patients.

	Patients with PE (n = 32)	Control Group (n = 32)	*p*
Age ^a^	32.4 ± 6.7	29.2 ± 6.6	>0.05
Gravida ^b^	3 (1–7)	2 (1–7)	>0.05
Parity ^b^	1 (0–4)	0 (0–5)	>0.05
BMI (kg/m^2^) ^a^	30.09 ± 4.9	28.6 ± 3.4	>0.05
Gestational age at recruitment (weeks) ^b^	32 (26–36)	33 (29–36)	>0.05
Gestational age at delivery (weeks) ^b^	34 (26–37)	38 (37–41)	**<0.001**
Cesarean section ^c^	27 (84.4%)	23 (71.8%)	>0.05
Neonatal gender, male ^c^	15 (46.9%)	14 (43.8%)	>0.05
Neonatal birth weight (g) ^a^	1941.83 ± 1043.58	3221.30 ± 526.03	**<0.001**
5 min APGAR score ^b^	7 (1–10)	9 (4–10)	0.16

BMI: Body Mass Index, ^a^ mean ± standard deviation, ^b^ median (range values in parentheses), ^c^ number of cases (percentages in parentheses). The bold *p* values indicated the statistically significant values.

**Table 2:** Comparison of maternal serum IMA, SCUBE-1, and Doppler findings of the cases.

	Patients with PE (n = 32)	Control Group (n = 32)	*p*
Serum IMA (ABSU)	0.80 ± 0.13	0.73 ± 0.67	**0.004**
Serum SCUBE-1 (ng/mL)	2.84 ± 1.11	1.69 ± 0.53	**0.000**
UA-PI	1.13 ± 0.29	0.93 ± 0.12	**0.001**
MCA-PI	1.49 ± 0.41	1.62 ± 0.29	0.152
CPR	1.41 ± 0.49	1.75 ± 0.32	**0.002**
Right UtA-PI	1.29 ± 0.54	0.65 ± 0.20	**0.000**
Left UtA-PI	1.18 ± 0.49	0.68 ± 0.18	**0.000**

ABSU: Absorbance unit, IMA: Ischemia modified albumin, SCUBE-1: Signal peptide-CUB (complement C1r/C1s, Uegf, and Bmp1, UA-PI: Umbilical artery pulsatility index, MCA-PI: Mean cerebral artery-Pulsatility index, CPR: Cerebroplacental ratio, UtA-PI: Uterine artery pulsatility index. Student’s t-test was used for comparison. The bold *p* values indicated the statistically significant values.

**Table 3:** Correlation analysis of maternal serum IMA, serum SCUBE1 levels and maternal–fetal Doppler findings.

	UA-PI		CPR		Right UtA-PI		Left UtA-PI		Neonatal Weight	
	*r*	*p*	*r*	*p*	*r*	*p*	*r*	*p*	*r*	*p*
Serum SCUBE-1	0.147	0.245	−0.188	0.136	0.360	0.004	0.340	0.006	−0.350	0.08
Serum IMA	0.180	0.154	−0.220	0.08	0.199	0.115	0.54	0.674	−0.401	0.002

IMA: Ischemia modified albumin, SCUBE-1: Signal peptide-CUB (complement C1r/C1s, Uegf, and Bmp1, UA-PI: Umbilical artery pulsatility index, CPR: Cerebroplacental ratio, UtA-PI: Uterine artery pulsatility index, r: correlation coefficient, p: statistical significance value. Spearman correlation test was used.

## Data Availability

Any dataset is available on request to the corresponding author.
